# Effect of structure and composition of cationic liposomes on the delivery of siRNA *in vitro* and *in vivo*

**DOI:** 10.3389/fphar.2025.1656671

**Published:** 2025-09-12

**Authors:** Daniil V. Gladkikh, Elena V. Shmendel, Darya M. Makarova, Mikhail A. Maslov, Marina A. Zenkova, Elena L. Chernolovskaya

**Affiliations:** ^1^ Institute of Chemical Biology and Fundamental Medicine SB RAS, Novosibirsk, Russia; ^2^ Lomonosov Institute of Fine Chemical Technologies, MIREA – Russian Technological University, Moscow, Russia

**Keywords:** liposomes, delivery of siRNA, lipoconjugate, biodistribution, TTR gene silencing

## Abstract

This study explores the effects of the modifications of siRNA delivery systems based on cationic liposomes containing the polycationic amphiphile 2 × 3 and lipid-helper DOPE on their ability to deliver therapeutic siRNA *in vitro* and *in vivo*. We supplied the core liposome system with lipoconjugates differing in PEG length weights and conjugate structure, and additionally modified with a folate residue as an addressing moiety. The *in vitro* data revealed no direct correlations between PEG length, lipoconjugate structure and the transfection efficiency of siRNA lipoplexes. *In vivo* biodistribution studies highlighted the significant influence of tumor presence on siRNA accumulation and biodistribution, underscoring the importance of adaptive delivery systems. In healthy mice, the largest amount of siRNA accumulates in the liver, whereas in tumor-bearing mice, accumulation in the kidneys increases, with a noticeable amount of siRNA accumulating in the tumor. Despite the longer linear PEG increasing the circulation time of siRNA, diP800 showed the best tumor accumulation. Anti-TTR siRNA complexes with all liposomal formulations demonstrated significant suppression of the *Ttr* mRNA in the liver, complexes with diP2000 and 2 × 3 liposomes demonstrated the highest silencing efficiency. These results contribute to advancing nucleic acid therapeutics by offering a comprehensive understanding of liposomal delivery system optimization.

## 1 Introduction

In recent years, the field of pharmaceutical research has witnessed significant advancements in the development of drug delivery systems tailored to enhance the efficacy of various therapeutic agents. Among these agents, small interfering RNA (siRNA) has emerged as a promising tool for targeted gene silencing, with potential applications ranging from cancer treatment to the management of genetic disorders. FDA has already approved six siRNA-based drugs: patisiran (2018), givosiran (2019), lumasiran (2020) (May Zhang et al., 2021), inclisiran (2021) (Padda et al., 2024), vutrisiran (2022) (Keam S.J., 2022), and nedosiran (2023) (Liu et al., 2022; Syed, 2023). At least three siRNA-based drugs (fitusiran (Young et al., 2023), teprasiran (Thielmann et al., 2021), and tivanisiran (Traber and Yu, 2023)) are in the different late stages of phase two to three clinical trials, some of them are very close to market release. However, the clinical translation of siRNA-based therapeutics is hampered by challenges related to their delivery to specific target cells and tissues. Naked siRNA molecules are rapidly degraded in the bloodstream, have limited cellular uptake, and face obstacles in traversing cellular membranes. Furthermore, specific targeting of disease-associated cells while sparing healthy tissues remains an unsolved problem (Smith et al., 2022). Current methods of siRNA delivery include viral vectors, bioconjugation, lipid-based systems, and polymer-based carriers. Viral vectors offer high efficiency but pose risks of immunogenicity and insertional mutagenesis (Lawler et al., 2017). Bioconjugation has been successfully used to deliver therapeutic siRNAs to liver cells, five out of six drugs used in the clinic are bioconjugates, but effective biomolecules for delivering siRNA to extrahepatic tissues have not yet been found (May Zhang et al., 2021). Polymer-based systems can be tailored for specific applications but often suffer from toxicity and complex manufacturing processes (Singha et al., 2011). Lipid-based delivery systems, particularly lipid nanoparticles (LNPs), have emerged as a leading approach for siRNA delivery. These systems are attractive due to their biocompatibility, ability to encapsulate siRNA, and facilitate its endosomal escape. LNPs can protect siRNA from degradation, enhance cellular uptake, and enable targeted delivery through surface modification with cell-specific ligands. However, despite these advantages, lipid-based systems still face limitations such as potential immunogenicity, off-target effects, and challenges in large-scale production (Samaridou et al., 2020). The strategic incorporation of specific ligands on the surface of lipid-based nanoparticles is anticipated to address some of the persistent challenges in the field of siRNA therapeutics and pave the way for more effective and safer treatments (Gladkikh et al., 2021). Consequently, effective delivery systems that can protect, transport, and release siRNA at the intended site of action are of paramount importance.

Previously, we developed and studied a set of liposomal formulations for the delivery of nucleic acids into the cells based on the polycationic amphiphile 1,26-bis (cholest-5-en-3-yloxycarbonylamino)-7,11,16,20-tetraazahexacosane tetrahydrochloride (2 × 3) (Petukhov et al., 2010) which has the ability to electrostatically interact with nucleic acids and condense it into nanoparticles, protecting them from degradation and facilitating better penetration into tumor cells to address this challenge. These liposomal formulation due to the presence of lipid-helper 1,2-dioleoyl-*sn*-glycero-3-phosphoethanolamine (DOPE) provides effective endosomal escape of various nucleic acids including siRNA, which is critical for its biological activity (Maslov et al., 2012; Kabilova T. O. et al., 2018). PEG lipoconjugates have been frequently employed to modify siRNA delivery systems. PEGylation confers several advantages, including increased stability, prolonged circulation time, and reduced immunogenicity. Tailoring PEG modifications in terms of molecular weight and structure can fine-tune the pharmacokinetics and biodistribution of siRNA carriers (Kabilova T. et al., 2018). The incorporation of targeting ligands, such as folate, provides a means to achieve site-specific delivery of siRNA. Folate receptors are often overexpressed on the surface of cancer cells, making folate a promising ligand for targeted therapy. Utilizing folate as a targeting component offers the potential to enhance the selectivity of siRNA delivery, thus reducing off-target effects and minimizing side effects (Kabilova T. et al., 2018). We have investigated the effect of cationic liposome composition on the performance of cargo immunostimulatory RNA (Bishani et al., 2023) and its ability to limit influenza infection in C57Bl/6 mice (Goncharova et al., 2020). However, the influence of the composition and structure of liposomes based on the 2 × 3-DOPE core system on the delivery and biological activity of siRNA *in vitro* and *in vivo* has not been systematically studied previously.

In this study, specifically, we examine the impact of variations in the molecular weights and structures of PEG lipoconjugates, as well as the inclusion of folate as a targeting component, on the performance of the delivery system. Our evaluation of efficacy encompasses comprehensive assessments in both *in vitro* and *in vivo* settings, providing a holistic understanding of the delivery system’s capabilities.

## 2 Materials and methods

### 2.1 Liposomes synthesis and preparation

**FIGURE 1:**
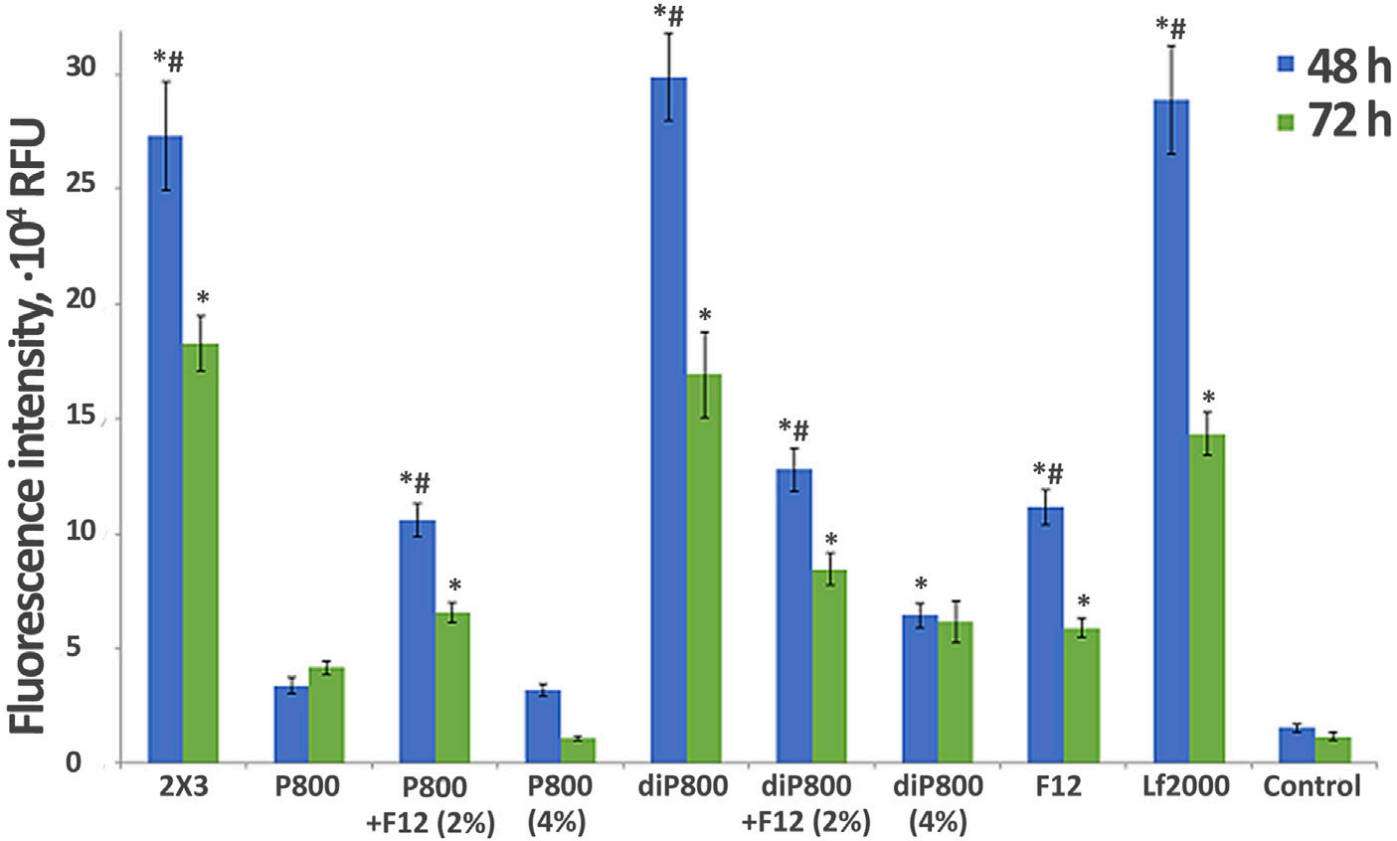
Effect of liposome composition on the efficiency of *in vitro* delivery of Cy-5.5-labeled siRNA into the KB-3-1 cell at different N/P. Flow cytometry data (RFU - Relative Fluorescence Units), 4 h after transfection in the serum-free medium (blue bars) or medium with 10% FBS (red bars) (n = 3, t-test, *p ≤ 0.01, **p ≤ 0.05 the value in serum free medium compared to same lipoplex with the same N/P in the presence of serum).

The following liposome components were synthesized as previously described: 2 × 3 (Maslov et al., 2012), PEG conjugates with one anchor group *O*-(2-(acet)amidoethyl)-*O*′-[2-(*rac*-2,3-di(tetradecyloxy)prop-1-yloxycarbonyl)aminoethyl] octadecaethylene glycol (P800), *O*-(3-(acetamidopropyl)-*O*′-[3-(*rac*-2,3-di(tetradecyloxy)prop-1-yloxycarbonyl)aminopropyl] poly (ethylene glycol_1500_) (P1500), *O*-(2-(acetamidoethyl)-O′-[2-(rac-2,3-di(tetradecyloxy)prop-1-yloxycarbonyl)aminoethyl] poly (ethylene glycol_2000_) (P2000) (Kabilova T. et al., 2018), similar PEG conjugates with two anchor groups *O*,*O*′-bis [*rac*-2,3-di(tetradecyloxy)propyl-1-oxycarbonylamino] octadecaethylene glycol (diP800), *O*,*O*′-bis [*rac*-2,3-di(tetradecyloxy)propyl-1-oxycarbonylamino] poly (ethylene glycol_1500_) (diP1500), *O*,*O*′-bis [*rac*-2,3-di(tetradecyloxy)propyl-1-oxycarbonylamino] poly (ethylene glycol_2000_) (diP2000) (Bishani et al., 2023), and PEG (800 Da) conjugate equipped with folate residue F12 (Kabilova T. O. et al., 2018). All liposomal formulations were prepared as previously described (Maslov et al., 2012) and the structures of the compounds included in liposomes are presented in Figure 1.

### 2.2 Synthesis of siRNAs and siRNA/liposome complexes preparation

The synthesis of the 2′-O-methylated siTTR (sense strand 5′- CmAGUmGUUfCUfUfGfCUCUmAUmAAUU-3'; antisense strand 5′- UUfAUmAGfAGCmAAGAAfCmAfCUmGUU-3′) and siScr (sense strand 5′- CCmACUmACmAUmACGAGACUUmGUU-3′; antisense strand 5′-CmAAGUCUCGUmAUmGUmAGUmGGU-3′) used in this study was carried out as previously described in our earlier works (Chernikov et al., 2023). Cyanine 5.5 (Cy5.5) were attached to siScr through a 3′-aminohexyl linker according to the manufacturer’s protocol, using Cy5.5 *N*-hydroxysuccinimide ester (Biotech Industry Ltd., Moscow, Russia) in 0.1 M Tris buffer (pH 8.4). Strands of siRNAs (50 μM) were annealed in a buffer containing 30 mM HEPES-KOH (pH 7.4), 100 mM sodium acetate, and 2 mM magnesium acetate, by heating at 90 °C for 5 min, followed by cooling to room temperature. The siRNA preparations were stored at −20 °C until use. For the *in vivo* studies, the cationic liposomes and siRNA complexes (at N/P ratio 4/1 of positively-chargeable polymer amine (N) groups to negatively-charged nucleic acid phosphate (P)) were formed in a volume of 200 µL by mixing 100 µL siRNA (final concentration of 7 µM) and 100 µL liposome at corresponding concentration. For the *in vitro* studies, the cationic liposomes and siRNA complexes at different (2/1, 4/1, 8/1) N/P ratios were formed in a volume of 200 µL by mixing 100 µL siRNA (final concentration of 7 µM) and 100 µL liposome at corresponding concentration. Different N/P ratios were used based on preliminary optimization for each type of experiment. The N/P ratio of 8/1 provides maximum transfection efficiency *in vitro*, which is critical for assessing siRNA delivery potential. For *in vivo* studies, an N/P ratio of 4/1 was used because higher ratios may cause toxic effects upon systemic administration, while this ratio provides optimal balance between efficacy and safety (Gladkikh et al., 2021). All solutions were prepared in serum-free OptiMEM (Invitrogen, Waltham, MA, USA). The resulting mixtures were incubated for 20 min at room temperature before intravenous (IV) administration to mice or before being added to the cells.

### 2.3 Liposome sizes, zeta potentials and siRNA binding analysis

Particle size and zeta potential were assessed using a Zetasizer Nano ZS (Malvern Panalytical Ltd., Malvern, UK). The average hydrodynamic diameter was determined from particle number distributions, and these measurements were repeated three times. For this, 50 µL of siRNA solution prepared in Opti-MEM was mixed with 50 µL of liposome solution in Opti-MEM taken at the appropriate N/P ratio. After incubating at room temperature for 20 min, the size analysis was conducted using a 100 µL microcuvette. To determine the zeta potential, 900 µL of MilliQ water was added to the sample, and the surface potential was recorded in a 1 mL cuvette, and these measurements were repeated three times. siRNA binding efficiency to liposomes was assessed using an agarose gel retardation assay. siRNA/liposome complexes at various N/P ratios were incubated for 20 min at room temperature, then analyzed by electrophoresis in 2% agarose gel with ethidium bromide (20 min, 120V). The percentage of bound siRNA was calculated as (1 - free siRNA intensity/control siRNA intensity) × 100%.

### 2.4 Animals

Adult male CBA and C57BL/6 mice (weighing 20–24 g) were obtained from the Center for Genetic Resources of Laboratory Animals at the Institute of Cytology and Genetics SB RAS. These animals were housed in the vivarium of the Institute of Chemical Biology and Fundamental Medicine, SB RAS, under natural light conditions and provided with a standard laboratory diet, adhering to international guidelines for proper use and care of laboratory animals (ECC Directive 86/609/EEC). The experimental protocol was approved by the Committee on the Ethics of Animal Experiments at the Institute of Cytology and Genetics of SB RAS (protocols #22.11 from 30.05.2014 and #220 from 06.03.2025).

### 2.5 Tumor cell lines

Human cervical carcinoma KB-3-1 cells were obtained from the Russian Cell Culture Collection at the Institute of Cytology, Russian Academy of Sciences, St. Petersburg, Russia. The cells were cultured in DMEM (Sigma-Aldrich, St. Louis, MO, USA) supplemented with 10% FBS (HyClone, GE Healthcare, Chicago, IL, USA) and 1% antibiotic-antimycotic solution (MP Biomedicals, Santa Ana, CA, USA). The RLS_40_ drug-resistant murine lymphosarcoma was previously developed from the chemotherapy-susceptible lymphosarcoma LS at this Institute (Mironova et al., 2006). RLS_40_ cells were cultured in IMDM (Sigma-Aldrich, St. Louis, MO, USA) supplemented with 10% FBS, 1% antibiotic-antimycotic solution, and 40 nM vinblastine. All cells were grown in a humidified environment with 5% CO_2_ and 95% air at 37 °C and were regularly passaged to maintain exponential growth.

### 2.6 Cellular accumulation assay

One day prior to the experiment, KB-3-1 cells in the exponential growth phase were plated onto 48-well plates at a density of 5.5 × 10^4^ cells per well. For serum-containing conditions, Cy5.5-labeled siScr or its complexes with liposomes were added to the cells after a 24-h incubation period. For serum-free conditions, the medium was changed to serum-free medium before adding siRNA. Four hours after the addition of Cy5.5-siScr, cells were detached using TrypLE (Thermo Fisher Scientific, Waltham, MA, USA) and fixed in 2% formaldehyde in phosphate-buffered saline (PBS). Cell analysis was conducted using a NovoCyte flow cytometer (ACEA Biosciences); 15,000 cells from each sample were analyzed.

### 2.7 *In Vivo* biodistribution studies

*In vivo* real-time fluorescence imaging was employed to assess the distribution of Cy5.5-labeled siScr in healthy and tumor-bearing mice. The IVIS Lumina X5 Imaging system (Perkin Elmer, Waltham, Massachusetts, USA) was used to capture X-ray and simultaneous near-infrared fluorescence (NIRF) images (Cy5.5: excitation 683 nm, emission 703 nm). Mouse RLS_40_ tumors were induced in CBA mice as previously described and allowed to grow to approximately 1,000 mm^3^ in size (Mironova et al., 2006; Mohamed et al., 2020). Mice (two mice per group) were IV administered with 10 µg/mice of Cy5.5-siScr complexed with cationic liposomes in 200 μL of OptiMEM prior to measuring. The animals were anesthetized with Isoflurane and placed on a heating tray (37 °C). Fluorescence scans (10 s exposure) and X-ray scans (15 s exposure) were registered 24 h after injection. Upon completion of full-body imaging, the mice were humanely euthanized, and tissues including the brain, lung, heart, liver, spleen, kidney, and tumors were harvested for subsequent analysis. Each organ was rinsed with PBS, and the fluorescence intensity was measured. Subsequently, the fluorescence intensity from both tumors and organs was quantified by establishing an automatic region of interest (ROI) with a threshold set at 30% of the maximum intensity of each sample. The mean intensity of that area was determined by multiplying each luminance level by the number of pixels at that level and then dividing by the total number of gray levels. Fluorescence intensities were standardized by taking into account the peak angle of detection and the organ’s surface area. Images were exported in batches as 16-bit TIFF files, and overlays, cropping and lettering were created using Adobe Photoshop CS3 (Adobe, San Jose, CA, USA). Three independent sets of experiments with identical experimental settings were conducted. The use of two animals per group represents a preliminary screening to identify the most promising formulations. This approach aligns with the 3R principles (replacement, reduction, refinement) and allows minimizing the number of experimental animals at the primary selection stage. The obtained data showed high convergence of results between mice in the group.

### 2.8 Quantitative stem-loop real-time PCR analysis dynamics of siRNA concentration in blood plasma

For plasma preparation 50 µL blood samples were collected for from CBA mice 15, 60 and 120 min after IV injection of 0.5 μg/g siTTR/liposome complexes (N/P = 4/1). The RNA from plasma samples was isolated by heating-in-Triton (Landesman et al., 2010). All siRNA specific stem-loop primers for qRT-PCR analysis were designed according to the instructions of Czimmerer (Czimmerer et al., 2013) using UPL-probe based stem-loop quantitative PCR assay design software (Astrid Research, Debrecen, Hungary) (http://genomics.dote.hu:8080/mirnadesigntool).

The reverse transcription reaction was performed in a final volume of 40 µL containing 4 µL of Triton X-100 preheated plasma supernatant, 8 µL 10 µM siTTR specific stem loop-RT primer (5′- GTT​GGC​TCT​GGT​GCA​GGG​TCC​GAG​GTA​TTC​GCA​CCA​GAG​CCA​ACA​ATT​AT -3′) 8 µL sterile water and 20 µL of Master Mix prepared from a M-MuLV–RH Reverse Transcription Kit (Biolabmix, Novosibirsk, Russia). The reaction mixture was incubated at 42 °C for 1 h. The cDNA was amplified in a total volume of 50 µL containing 10 µL of cDNA template, 10 µL siTTR specific forward (5′-GTG​GCA​GTG​TTC​TTG​CTC​T-3′) and reverse (5′- GTG​CAG​GGT​CCG​AGG​T-3′) primers (1 µM) and 5 µL sterile water and 25 µL of BioMaster HS-qPCR SYBR Blue master mix with SYBR Green I fluorescent dye (Biolabmix, Novosibirsk, Russia) using an CFX96 Real-time system (Bio-Rad, Hercules, CA, USA) according to the following scheme: one cycle—5 min, 95 °C; 35 cycles—10 s, 95 °C; 30 s, 51 °C; and 30 s, 72 °C. All measurements were done in triplicate.

### 2.9 Assessment of biological activity

Biological activity was determined by qRT-PCR analysis by the level of silencing of the expression of *Ttr* gene in the C57BL/6 mice liver under the action of siTTR 7 days after its IV administration in complexes with liposomes - the percentage was calculated relative to the activity after intravenous administration of scrambled siScr complexed with the same liposomes. Liver samples were isolated by heating-in-Triton (Landesman et al., 2010). cDNAs were synthesized using RT buffer and M-MuLV-RH revertase (Biolabmix, Novosibirsk, Russia) according to the manufacturer’s instructions and dT15 primer. Real-time PCR was carried out using an CFX96 Real Time System (Bio-Rad Laboratories Inc., Hercules, CA, USA) according to the following scheme: one cycle—5 min, 95 °C; 40 cycles—10 s, 95 °C; 40 s, 56 °C. All measurements were carried out in triplicate. Normalization was performed on the HPRT gene. The following primers were used:

HPRT Forward 5′- CCC​CAA​AAT​GGT​TAA​GGT​TGC - 3′,
HPRT Reverse 5′- AAC​AAA​GTC​TGG​CCT​GTA​TCC - 3′,
HPRT Probe 5’- ((5,6)-ROX) - CTT​GCT​GGT​GAA​AAG​GAC​CTC​TCG​AA - BHQ2 - 3′,
mTTR Forward 5′- AAT​CGT​ACT​GGA​AGA​CAC​TTG​G - 3′,
mTTR Reverse 5′- TGG​TGC​TGT​AGG​AGT​ATG​GG - 3′, mTTR Probe 5’- ((5,6)-FAM) - AGG​GCT​GCG​AAT​GGT​GTA​GTG​G - BHQ1 - 3’.

The relative level of gene expression was calculated using the Bio-Rad CFX software (Bio-Rad Laboratories Inc., Hercules, CA, USA).

### 2.10 Statistical analysis

Statistical analysis was conducted using GraphPad Prism 8.4.3 software (GraphPad Software, Inc., San Diego, CA, USA), and the data is presented as the mean ± standard deviation (SD). Statistically significant differences were determined using an ordinary two-way ANOVA with Dunnett’s multiple comparisons test. The differences between the values are considered statistically significant when p < 0.05.

## 3 Results

### 3.1 The key characteristics of the prepared liposomes and lipoplexes

In this work, we used a series of cationic liposomes based on the core 2 × 3-DOPE system (here and after 2 × 3) which, as shown in our previous work, has already proven itself to be effective for the delivery of siRNA *in vitro* and *in vivo* (Maslov et al., 2012; Kabilova T. et al., 2018; Kabilova T. et al., 2018). The aim of our study was to determine how the addition of different lipoconjugates (Supplementary Figure S1) to the formulation affects the ability of liposomes to deliver siRNA and provide its biological activity. We prepared a set of liposomes (Table 1) with a 1:1 ratio of 2 × 3 to DOPE since it has been shown that this ratio is optimal for siRNA delivery *in vivo* (Kabilova T. O. et al., 2018). The liposomes were modified with PEG conjugates of various molecular weights (800, 1,500, and 2000 Da) to stabilize the liposomes and their complexes with siRNA in the blood stream. PEG lipoconjugates of two different structures were used: linear, containing one anchor group (P series) and loop-like, containing two identical anchor groups at the ends of the PEG chain (diP series). The latter configuration allows for the formation of a denser yet thinner protective shell. We have previously used this liposome array to deliver immunostimulatory RNA (Bishani et al., 2023), but it has not been studied for siRNA delivery.

**TABLE 1:** The composition and characteristics of the cationic liposomes used in the study and their complexes formed with siRNA.

Liposome	Composition, mol%	N/P	Size. d Nm	Z. mV	PdI
2 × 3-DOPE	2 × 3/DOPE (50/50)	-	93.5 ± 1.9	45.8 ± 1.1	0.4
		2/1	98.2 ± 2.1	30.2 ± 2.1	0.2
		4/1	99.7 ± 3.1	24.1 ± 2.0	0.2
		8/1	167.2 ± 12.9	34.5 ± 1.1	0.5
P800	2 × 3/DOPE/P800 (49/49/2)	-	99.8 ± 3.1	40.8 ± 2.9	0.5
		2/1	120.2 ± 4.2	32.7 ± 1.9	0.4
		4/1	158.7 ± 6.2	24.3 ± 3.5	0.4
		8/1	253.3 ± 12.0	37.7 ± 1.4	0.3
P800 + F12 (2%)	2 × 3/DOPE/P800/F12 (48/48/2/2)	-	57.9 ± 1.0	40.6 ± 2.4	0.3
		2/1	124.4 ± 2.2	34.6 ± 2.1	0.4
		4/1	221.5 ± 17.4	37.9 ± 2.5	0.4
		8/1	311.4 ± 19.3	38.4 ± 0.4	0.3
P800 (4%)	2 × 3/DOPE/P800 (48/48/4)	-	61.5 ± 2.1	40.7 ± 1.2	0.4
		2/1	108.6 ± 3.8	32.2 ± 1.0	0.4
		4/1	140.2 ± 10.1	35.3 ± 0.5	0.3
		8/1	284.5 ± 97.7	33.0 ± 3.0	0.4
diP800	2 × 3/DOPE/diP800 (49/49/2)	-	91.6 ± 1.1	52.8 ± 0.3	0.2
		2/1	162.1 ± 5.4	42.8 ± 2.4	0.3
		4/1	253.6 ± 8.2	39.6 ± 1.3	0.3
		8/1	162.9 ± 3.5	37.5 ± 0.7	0.4
diP800 + F12 (2%)	2 × 3/DOPE/diP800/F12 (48/48/2/2)	-	110.6 ± 2.5	47.7 ± 0.4	0.3
		2/1	172.2 ± 10.9	39.4 ± 1.2	0.4
		4/1	196.2 ± 16.7	40.8 ± 0.9	0.3
		8/1	169.7 ± 11.1	40.0 ± 1.0	0.4
diP800 (4%)	2 × 3/DOPE/diP800 (48/48/4)	-	127.3 ± 1.5	48.9 ± 0.9	0.5
		2/1	140.5 ± 3.8	41.7 ± 1.7	0.5
		4/1	256.7 ± 15.5	36.9 ± 1.9	0.4
		8/1	232.2 ± 20.9	36.6 ± 1.6	0.4
P1500	2 × 3/DOPE/P1500 (49/49/2)	-	102.0 ± 6.8	21.4 ± 2.1	0.3
		2/1	134.7 ± 7.1	25.2 ± 1.2	0.4
		4/1	179.6 ± 6.7	29.1 ± 1.4	0.4
		8/1	239.6 ± 34.0	29.9 ± 4.2	0.6
diP1500	2 × 3/DOPE/diP1500 (49/49/2)	-	98.0 ± 4.3	51.3 ± 1.1	0.4
		2/1	103.2 ± 3.3	22.1 ± 2.1	0.4
		4/1	127.4 ± 1.8	11.1 ± 1.2	0.4
		8/1	140.5 ± 2.3	9.2 ± 0.6	0.4
P2000	2 × 3/DOPE/P2000 (49/49/2)	-	67.4 ± 2.1	47.3 ± 1.3	0.4
		2/1	100.3 ± 2.4	32.3 ± 0.9	0.4
		4/1	128.4 ± 14.9	16.5 ± 4.3	0.5
		8/1	214.3 ± 4.3	3.2 ± 0.9	0.7
diP2000	2 × 3/DOPE/diP 2000 (49/49/2)	-	109.8 ± 1.2	54.1 ± 2.7	0.3
		2/1	148.9 ± 3.1	24.6 ± 1.5	0.3
		4/1	162.6 ± 4.0	15.7 ± 0.6	0.4
		8/1	159.6 ± 13.6	4.1 ± 1.1	0.4
F12	2 × 3/DOPE/F12 (49/49/2)	-	77.3 ± 9.5	35.7 ± 5.6	0.4
		2/1	189.5 ± 12.7	32.4 ± 9.6	0.5
		4/1	260.9 ± 4.4	35.8 ± 0.2	0.3
		8/1	366.8 ± 77.8	35.8 ± 1.2	0.4

The content of lipoconjugates in the liposomal formulation ranged from 2% to 4% to determine the level that provides the most effective protection against serum aggregation and opsonization by serum proteins. The density of the targeting ligand onto the surface of liposomes is a variable that can affect the liposome’s ability to bind to target cells and be internalized into them (Elias et al., 2013; Alkilany et al., 2019; Duskey et al., 2022). The choice of 2% and 4% is based on our previous studies (Kabilova T. et al., 2018; Goncharova et al., 2020), which showed that these concentrations provide optimal balance between serum stability and transfection efficiency. Concentrations below 2% are insufficient for protection against serum proteins, while concentrations above 4% may reduce cellular uptake due to excessive charge shielding. Additionally, a folate-containing lipoconjugate (F12), composed of 1,2-di-*O*-ditetradecyl-*rac*-glycerol and folic acid linked through an 800 Da PEG spacer, was incorporated into liposomes either alone or in combination with other PEG-containing lipoconjugates. This addition aimed to facilitate the interaction of liposomes with folate receptors, which are expressed on various types of tumor cells (Parker et al., 2005; Siwowska et al., 2017; Kabilova T. O et al., 2018).

Notably, the core liposomes (2 × 3-DOPE) formed particles with an average size of approximately 93.5 nm (Table 1). The inclusion of lipoconjugates into the 2 × 3-DOPE composition resulted in variable effects on particle size depending on the lipoconjugate type: liposomes P800 + F12, P800 (4%), P2000 and F12 showed reduced size (to 57.9 nm, 61.5 nm, 67.4 nm and 77.3 nm, respectively), while P800, P1500, and diP series generally formed similar or slightly larger particles compared to the core liposomes.

Table 1 provides an overview of the characteristics of siRNA/liposome complexes formed at different N/P ratios (ranging from 2/1 to 4/1 and 8/1). These range provide a balance between efficacy and toxicity.

The particle sizes of empty liposomes and their complexes with siRNA are significantly affected by the lipoconjugate structure (linear P or loop diP) and PEG chain length (800, 1,500, 2000). Empty P800 liposomes have an average particle size of 99.8 nm, while diP800 liposomes exhibit a slightly smaller size of 91.6 nm, suggesting that the diP structure tends to yield slightly more compact particles in this context. However, when combined with siRNA, the particle sizes of P800 and diP800 diverge depending on the N/P ratio of the complex. For example, at a 4/1 ratio, P800 generates with siRNA particles with an average size of 158.7 nm, while diP800 gives larger particles 253.6 nm in size, indicating that diP800 tends to form larger complexes under these conditions. The difference in particle sizes between P1500 and diP1500 in their empty state is less pronounced compared to P800 and diP800. However, when complexed with siRNA at various ratios, P1500 and diP1500 exhibit substantial differences in particle size. For instance, at an 8/1 ratio, P1500 liposomes yield larger particles measuring 239.6 nm, while diP1500 liposomes create smaller particles of 140.5 nm. This highlights that diP1500 can form more compact complexes under specific conditions, in contrast to P1500. Empty P2000 and P800 + F12 liposomes exhibit the smallest particle size among the studied liposomes, measuring 67.4 and 57.9 nm, respectively, while diP2000 liposomes have a particle size of 109.8 nm. At lower N/P ratios (2/1 and 4/1), P2000 liposomes tend to form smaller complexes than diP2000 liposomes. However, at a higher N/P ratio (8/1), this trend reverses, with diP2000 forming more compact complexes (159.6 nm) compared to P2000 (214.3 nm). This suggests that the behavior of these formulations is highly dependent on the N/P ratio used. Noteworthy, the significant increase in the size of the complexes of P series liposomes with siRNA at an N/P ratio of 8/1 was detected, which does not occur in complexes formed by the diP series liposomes (except diP800 (4%)).

Liposomes containing lipoconjugates with looped PEG (diP series) typically exhibit a more positive zeta potential in empty form compared to the ones containing P series conjugates (with P2000 being an exception, showing a zeta potential of 47.3 mV, which is relatively close to the 54.1 mV of diP 2000). In the composition with siRNA, the zeta potentials of both series tend to be similar. As the length of polyethylene glycol molecules increases, the zeta potential decreases. This trend is observed for both linear and loop structures. Additionally, as expected, the complexation with siRNA often leads to a reduction in the zeta potentials of lipoplexes, and the polydispersity index (PDI) may vary depending on the structure and length of the molecules, generally increasing, indicating a greater diversity in molecule sizes or structural changes.

For P800 liposomes, augmentation of polyethyleneglycol lipoconjugate content to 4% results in the formation of significantly smaller particles compared to the same liposomes with 2% of lipoconjugate (from 99.8 nm to 61.5 nm) in their empty form. In contrast, for diP800 liposomes, increasing the lipoconjugate content to 4% leads to larger particles formation (from 91.6 nm to 127.3 nm) in their empty form. When complexed with siRNA at different ratios, the trend varies depending on the N/P ratio. For instance, at an N/P ratio of 2/1, both P800 and diP800 (4%) form smaller complexes (108.6 and 140.5 nm, respectively) than P800 and diP800 (2%) (120.2 and 162.1 nm, respectively). However, at higher N/P ratios (4/1 and 8/1), the effect becomes more complex and ratio-dependent. Liposomes containing 2% or 4% of PEG-lipoconjugate, as well as their complexes with siRNA, have similar zeta potentials. The PDI remains relatively stable across different ratios, indicating that the addition of additional amount of lipoconjugate has a minor impact on the homogeneity of particle size and shape for both P and diP structures.

The supplementation of 2% F12 to P800 results in a significant reduction in the size of empty liposomes (from 99.8 nm to 57.9 nm), while this moderately increases the size of empty diP800 liposomes (from 91.6 nm to 110.6 nm). Both P800 + F12 and diP800 + F12 show an increase in the particle sizes of their complexes with siRNA compared to their counterparts without F12. The addition of F12 appears to have an impact on zeta potential and PDI, depending on the structure and the presence of siRNA in the complex, but these changes seem to be of minor significance. siRNA binding analysis (Supplementary Figure S2) showed that all tested formulations demonstrate high complexation efficiency (>94%) at N/P ratio 4/1 and complete binding (>98%) at N/P 8/1. The presence of PEG-lipoconjugates did not affect siRNA binding efficiency, confirming the stability of electrostatic interactions between cationic lipids and siRNA. Addition of the folate ligand also does not interfere with the complex formation, indicating preservation of the carrier’s basic properties.

In summary, liposome structure, length, and PEG-lipoconjugate percentage significantly impact particle sizes, zeta potentials, and polydispersity indexes in both empty and siRNA-complexed forms. Loop diP structures generally exhibit more positive zeta potentials, with size effects varying based on structure and siRNA content. Higher PEG-lipoconjugate content reduces particle sizes, while the addition of F12 has minor effects. These insights are crucial for optimizing liposomal systems for therapeutic nucleic acids and provide a wide range of structures, among which optimal options for different applications can be found.

### 3.2 Accumulation of siRNA/liposome complexes in KB-3-1 cells

In the first stage of the study, we performed screening of the ability of liposomes containing 2% of different lipoconjugates to deliver siRNA to KB-3-1 cells *in vitro*. Lipoplexes were formed by mixing siRNA solution with cationic liposomes at different N/P ratio to examine the ability of the liposomes to transfer siRNA into the cultured cells. The choice of the appropriate N/P ratio is crucial for efficient siRNA compactization, negative charge neutralization and plays a vital role in transfection using liposomal formulations. We conducted experiments to analyze the intracellular accumulation of a Cy5.5-labeled siRNA mediated by liposomes containing 2% lipoconjugates under various conditions to identify the most effective liposomal formulation and the optimal composition of lipoplexes for siRNA delivery. In these experiments, KB-3-1 cells, which overexpress FR (Kabilova T. et al., 2018; Gladkikh et al., 2021), were transfected with siRNA/liposome complexes in the presence of 10% FBS or in the serum-free medium. Lipoplexes were formed at N/P ratios of 2/1, 4/1, and 8/1 for Cy5.5-siScr (0.2 µM final concentration). Flow cytometry was employed to assess the transfection efficiency of cationic liposomes, measured by the mean fluorescence intensity (MFI) of cells.

The obtained data show that under conditions used almost 100% of the cells were transfected, but the intensity of their fluorescence, corresponding to the amount of accumulated siRNA, differed, so we used this parameter (mean fluorescence intensity, MFI) to compare the effectiveness of liposomes (Figure 1). Based on the analysis, the optimal N/P ratio for Cy5.5-siScr delivery varies for each liposomal formulation and depends on transfection condition (with 10% serum or without it). However, for most lipoplexes, similar to 2 × 3 (Maslov et al., 2012), the transfection efficiency depends on the N/P ratio and increases with the increasing of the lipid content in the formulation. However, for some formulations such as diP1500, P1500, as well as P2000 and diP2000 in the serum-free medium, the differences in transfection efficiency observed at ratios of 4/1 and 8/1 are less pronounced compared to other formulations.

Among all tested formulations, the highest transfection efficiency was achieved using F12 at 8/1 ratio (∼8 × 10^4^ RFU), followed closely by 2 × 3 at the same ratio (∼7 × 10^4^ RFU). These formulations provide the highest fluorescence values particularly at 8/1 ratio, which is consistent with previously obtained data (Maslov et al., 2012). It should be noted that the effectiveness of 2 × 3 formulation is not sensitive to the presence of serum across all tested N/P ratios, whereas for F12, the serum independence is primarily observed at the high 8/1 ratio. At the same time, the structure and length of the PEG chain (800-1,500–2000) also influence the delivery efficiency.

Comparison of the level of siScr intracellular accumulation mediated by various PEG-containing liposomes (Figure 1) show that at low N/P 2/1 all liposomes regardless transfection conditions ( ±serum) provided similar transfection efficiencies (here and after TE). At N/P 4/1 and 8/1 huge differences between the formulations are observed depending on PEG length and presence of serum: high TE was observed for P800 (4/1, −serum), diP800 (4/1, −serum) < diP800 (8/1, ±serum), P1500 (4/1, 8/1, -serum), P2000 (8/1, +serum), diP 2000 (8/1, +serum). Conversion of the lipoconjugate structure from linear P to loop diP results in different impacts on the performance of the liposomes with different PEG lengths. Specifically, the corresponding liposomes of the diP800 series were more active than P800, while diP1500 was less active than P1500 and diP2000 was comparable in activity to P2000 in serum-free medium (Figure 1).

The obtained data showed that the majority of the studied liposomes exhibited higher transfection activity in the serum-free medium. The effectiveness of 2 × 3 formulation was relatively independent of the presence of serum. Interestingly, formulations like P2000 at the 4/1 and 8/1 ratios as well as diP2000 at 8/1 showed increased TE in the presence of 10% serum, which makes these formulation promising candidates for *in vivo* use.

Thus, the influence of PEG length, lipoconjugate structure, and transfection conditions on TE is complex and requires experimental verification, since there are no direct correlations. PEG length, conjugate structure, and the presence of serum can have both positive and negative effects on siRNA delivery, and the selection of optimal conditions and liposomes should be made depending on the specific objectives of the study.

Next, we performed experiments with P800 and diP800 liposomes to elucidate the impact of the lipoconjugate content in the formulation and/or the addition of another lipoconjugate on TE *in vitro*. These modifications included increasing the percentage of PEG lipoconjugate from 2% to 4%, and addition of the targeting component F12 (2% mol.) into the formulation. The duration of cell incubation after the addition of lipoplexes was increased to 48 and 72 h to assess the amount of siRNA in cells at time points when the maximum of its biological activity is expected to manifest.

**FIGURE 2:**
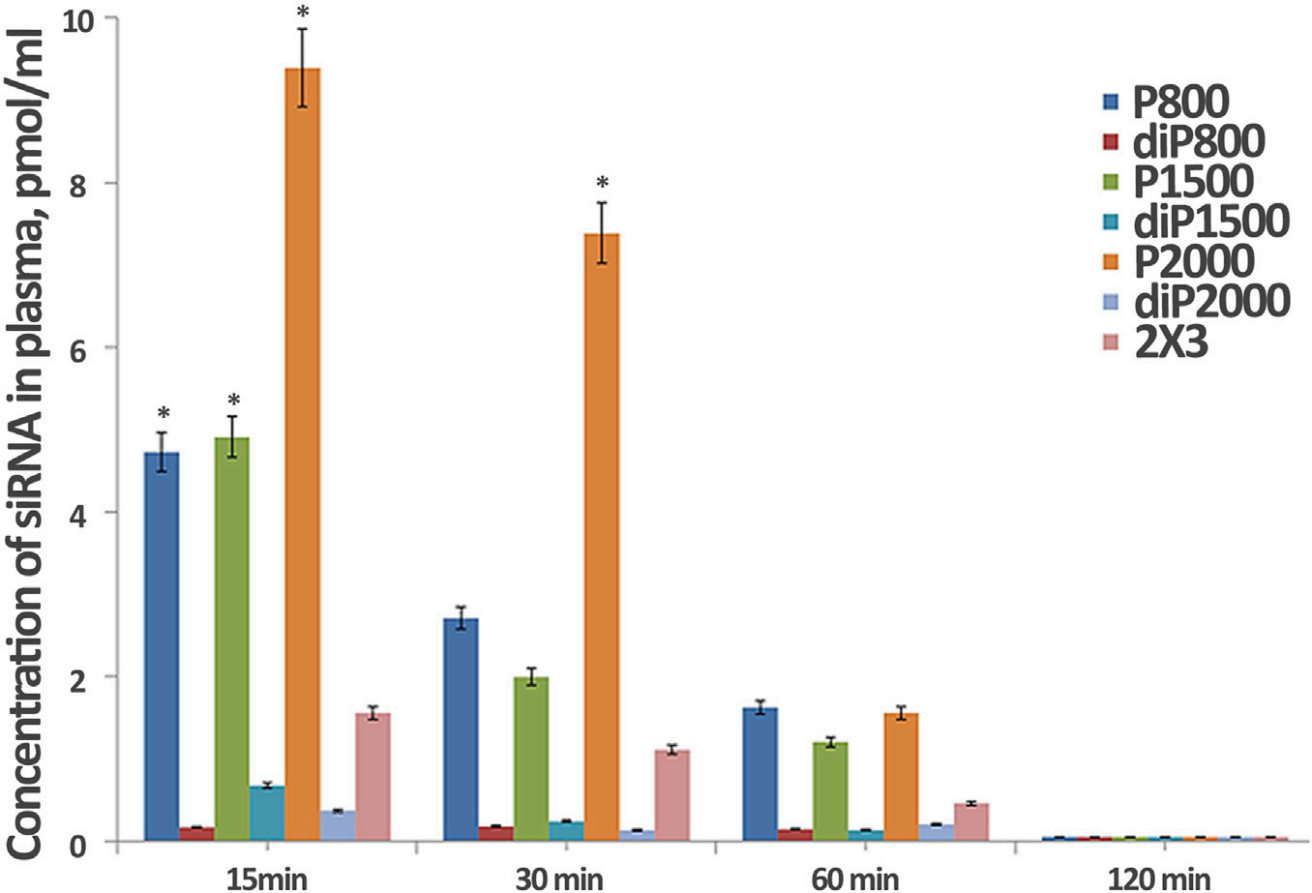
Effect of lipoconjugate content on the efficiency of *in vitro* delivery of Cy-5.5- siScr in KB-3-1 cells. Transfection was performed at N/P 8/1 for 4 h in serum-free medium, then serum was added to 10% and incubation was continued up to 48 and 72 h (n = 3, t-test, *p ≤ 0.01 compared to control PBS treated cells, #p ≤ 0.05 compared to 72 h).

Most formulations exhibit higher fluorescence intensity at 48 h compared to 72 h, indicating a peak in accumulation followed by a decline over time. The 2 × 3, diP800, and Lf2000 formulations demonstrate superior transfection efficiencies at both time points, consistently outperforming other tested formulations. Notably, diP800 shows the highest TE at 48 h, similar or somewhat exceeding that of the commercial transfection agent Lipophectamine 2000 (Lf 2000) and 2 × 3. Interestingly, the addition of F12 (2%) to P800 enhances its transfection efficiency compared to their initial formulations, suggesting a potential benefit of this modification. However, the same addition of F12 (2%) to diP800 demonstrates the opposite effect, significantly reducing its transfection efficiency by more than two-fold at 48 h and approximately two-fold at 72 h. This contrasting effect highlights the structure-dependent impact of folate targeting, where F12 benefits P800-based delivery but impairs the highly efficient diP800 system. However, increasing the content of P800 or diP800 from 2% to 4% does not significantly improve transfection efficiency and may even decrease it, indicating that higher lipoconjugate content does not necessarily correlate with better performance. The P800 formulation shows relatively low transfection efficiency compared to other formulations across both time points. Notably, all liposomal formulations demonstrate significantly higher transfection efficiency compared to the control at both 48 and 72 h, underscoring the overall effectiveness of these delivery systems for siRNA. The difference in the performance of P800 and diP800 after 4 h (Figure 1) and after 48–72 h (Figure 2) is striking: in the first case, the difference in transfection efficiency was insignificant, but over time, siRNAs delivered to cells using diP800 remain in much higher quantities.

### 3.3 Biodistribution of siRNA complexed with liposomes in healthy and tumor-bearing mice and TTR gene silencing in the liver, mediated by siTTR/liposome

The duration of siRNA circulation in the bloodstream, directly linked to the duration of drug interaction with target cells or tissues, is important for the development of effective drug delivery systems for systemic administration. This parameter is critical for the pharmacokinetics of drugs and the potential for adapting liposome properties to enhance therapeutic effectiveness and minimize side effects, as well as ensuring patient safety and treatment success.

We have previously shown that increased folate receptor-mediated accumulation of siRNA complexes with folate-containing liposomes in the cells and tumors expressing folate receptors (FR), is observed only at low N/P ratios of 1/1, 2/1 (Kabilova T. et al., 2018), whereas at high N/P they do not show an advantage of folate targeting compared to 2 × 3, which is consistent with our data (Figures 1, 2). Therefore, we did not include these liposomes in the *in vivo* studies.

**FIGURE 3:**
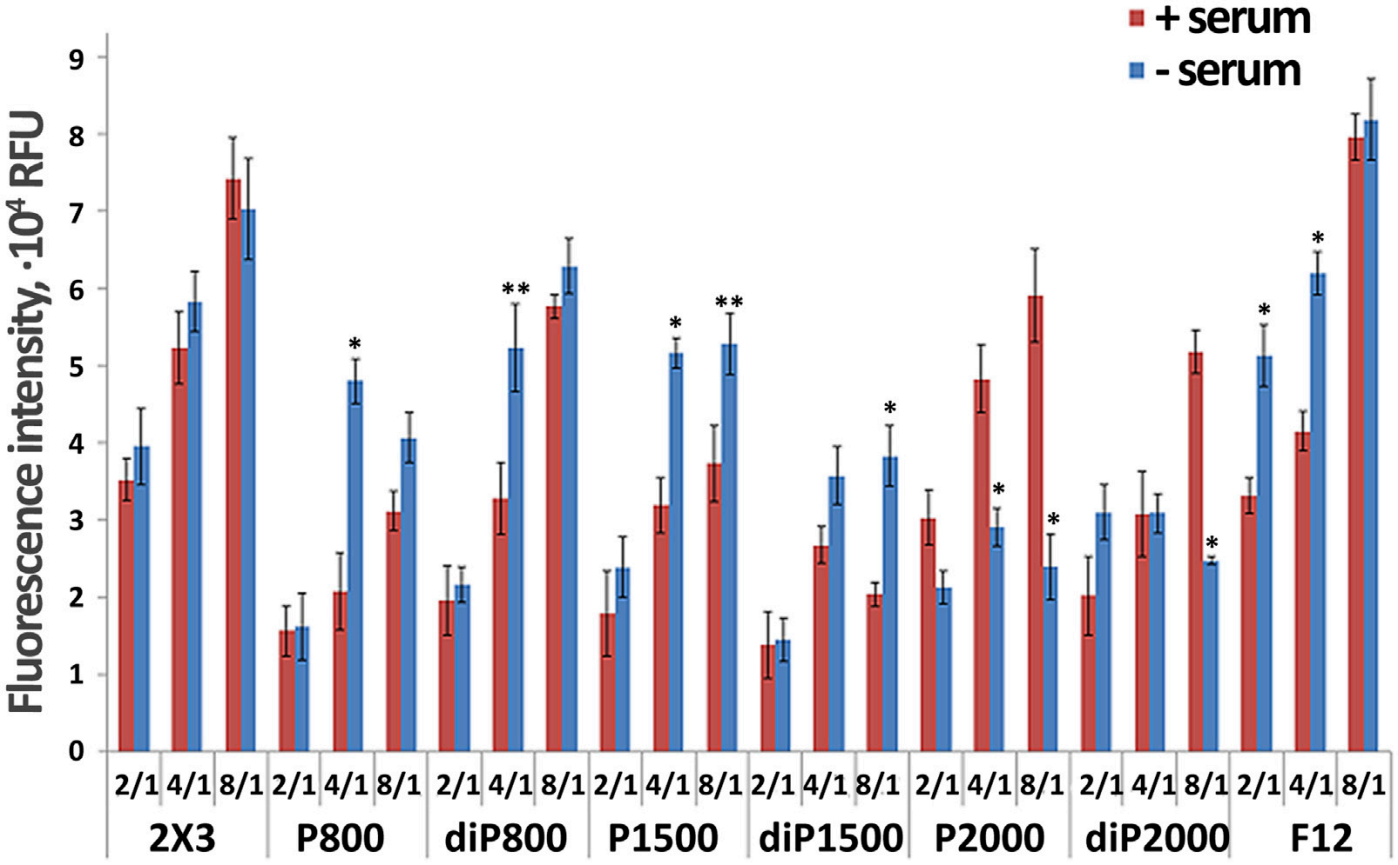
Dynamics of siRNA concentration in blood plasma of CBA mice after i. v. administration of its complexes with liposomes formed at N/P = 4/1 (*p ≤ 0.05 compared to lipoplexes with 2 × 3 at the same time point).

We examined the dynamics of siRNA concentration in mouse plasma after intravenous administration (IV) of siScr/liposome complexes by stem-loop PCR. The obtained data revealed that the composition and structure of liposomes affect their circulation time in the bloodstream (Figure 3). Complexes with liposomes of P—series, in general, exhibit longer circulation in the bloodstream compared to diP—series liposomes (Figure 3). siRNA/P2000 complexes maintain the highest siRNA concentration during the first 30 min after intravenous administration, but after 60 min, siRNA concentration decreased to the concentration of siRNA complexed with other P-series liposomes. Complexes of siRNA with diP liposomes behave differently from complexes with liposomes with liner PEG: they are quickly eliminated from the bloodstream and their concentration already at the first time point is significantly inferior those observed for P-series liposomes. The best results in diP series are observed for diP1500, however, the level of siRNA delivered by them is very low. Core liposomes 2 × 3 used as control maintain an intermediate concentration of siRNA in animal plasma compared to the liposomes of P and diP series, which gradually decreased to 60 min, but still remains on the levels much higher than that observed for diP-liposomes.

The biodistribution of Cy5.5-siScr in the major internal organs of healthy and tumor-bearing mice 24 h after IV administration of siRNA/liposome complexes was detected by fluorescence imaging using IVIS Lumina X5. In the first stage of the experiment, healthy CBA mice were IV injected with Cy5.5-siScr/liposome complexes, then after 24 h their internal organs were collected and Cy5.5 fluorescence was measured.

In this part of work, we did not investigate the biodistribution of siRNA complexes with 2 × 3 liposomes, as we have previously demonstrated their biodistribution patterns (Kabilova T. O. et al., 2018; Kabilova T. et al., 2018; Gladkikh et al., 2021) as well as ability to deliver short double stranded immunostimulating RNA (Bishani et al., 2023). These characteristics have already been well-established in our earlier studies, allowing us to focus on investigating alternative formulations in the current experiments. By building upon this foundation of knowledge regarding 2 × 3 performance, we were able to pay more attention to the comparative benefits of other liposomal formulations.

**FIGURE 4:**
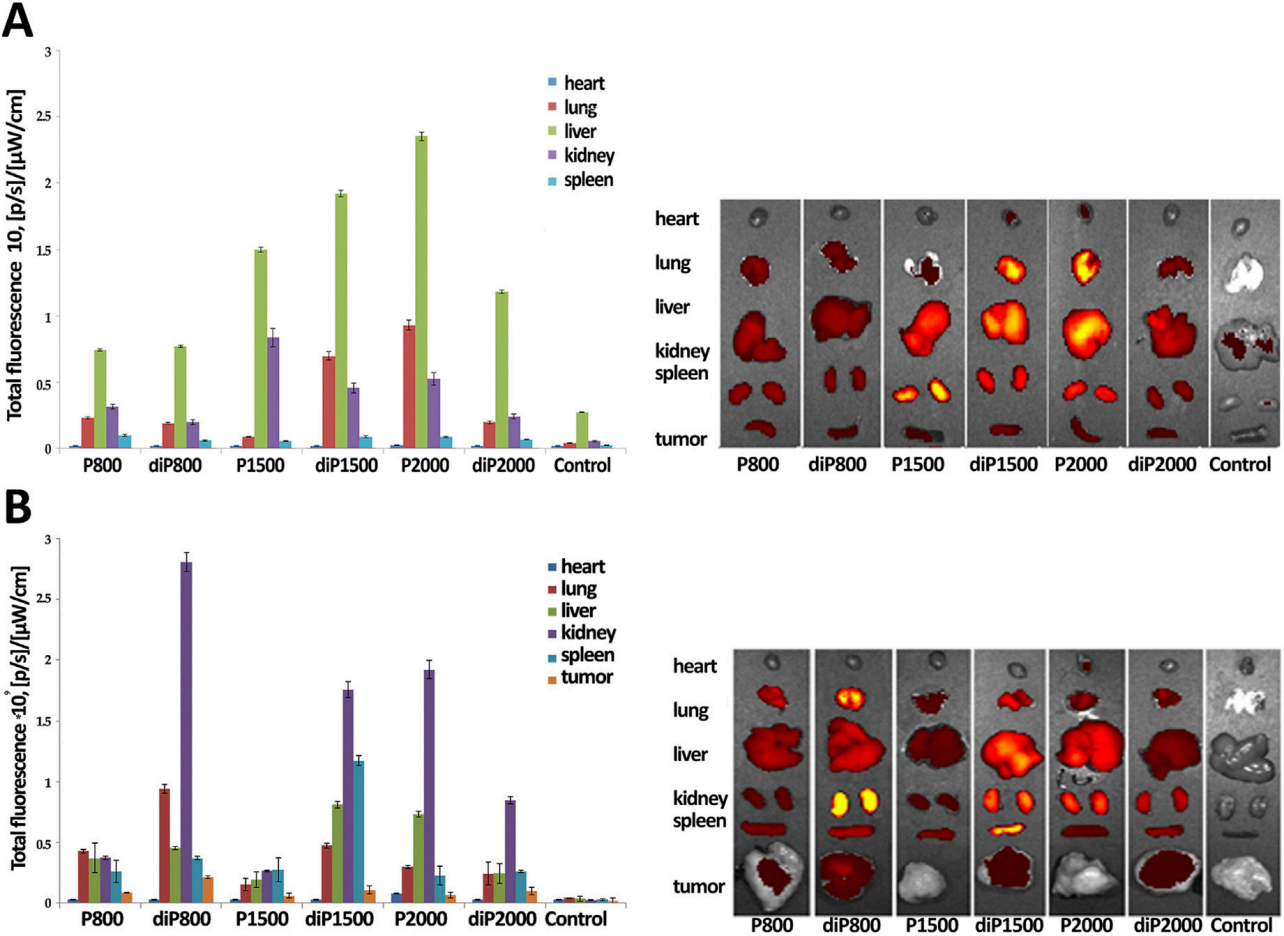
Biodistribution of Cy5.5-siScr lipoplexes in healthy **(A)** and RLS_40_ tumor-bearing **(B)** CBA mice. Images and fluorescence level of dissected main internal organs of CBA mice at 24 h after IV injection of the lipoplexes. Untreated mice were used as a control. N/P ratio 4/1.

The results presented in Figure 4A show that Cy5.5-siScr at N/P ratio 4/1 accumulates most efficiently in the liver of healthy mice; the highest levels of Cy5.5-siScr accumulation are achieved when liposomes P1500, diP1500 and P2000 were used. The kidneys also show significant Cy5.5-siScr accumulation mediated by the same liposomes. Elevated levels of Cy5.5-siScr accumulation in the lungs of healthy mice were recorded when siRNA was delivered in complexes with P2000 and diP1500: the level of its accumulation in the lungs was even higher than in the kidneys. The accumulation of siRNAs in other organs was insignificant.

In the second stage of the experiment, 10^6^ RLS_40_ cells in 0.1 mL of sterile saline buffer were implanted into the right thigh of CBA mice. Then, when tumor reached 1 sm^2^ in size, the mice were IV injected with Cy5.5-siScr/liposome complexes at N/P ratio 4/1 and after 24 h, the internal organs were collected and Cy5.5 fluorescence was measured (Figure 4B).

It is imperative to acknowledge that the presence of a tumor in the organism itself dramatically changes the accumulation and biodistribution of labeled Cy5.5-siScr in internal organs. The main difference concerns the balance of Cy5.5-siScr accumulation between the liver and kidneys: for complexes with diP800, diP1500, P2000 and diP 2000, accumulation in the kidneys of tumor-bearing mice increases significantly, while accumulation in the liver decreases. A decrease in Cy5.5-siScr accumulation in all studied organs of tumor-bearing mice was recorded for complexes with P1500. The increase in the accumulation of Cy5.5-siScr complexes with P800 and, especially, diP800 in the lung tissue with a slight decrease in the accumulation of complexes with diP1500 and P2000 also attracts attention, as well as the increased accumulation of diP1500 complexes in the spleen. The heart remains less affected by changes in Cy5.5-siScr accumulation in the presence of a tumor, but a slight decrease was also noted. In general, noticeable siRNA accumulation in the heart was observed only in the case of diP1500 and P2000 in animals without tumors, while in animals with tumors, accumulation was observed only for P2000 complexes.

It is noteworthy to emphasize that diP800 provides the best accumulation of siRNA in the tumor, accompanied by a higher fluorescence, while siRNA/P1500 accumulation in the tumor was the worst among the presented complexes. Thus, different liposomes should be used to achieve effective siRNA delivery to individual organs, given the fact that the health status of the animal may also influence the efficiency of accumulation. The biodistribution data provides no clear correlation in the efficiency of two groups of liposomes with PEG lipoconjugate structures and composition, which we observed during the analysis of data on the dynamics of siRNA concentration in blood plasma. However, it is important to note that the biodistribution of labeled siRNA does not necessarily directly correlate with the effectiveness of the gene silencing induced by delivered siRNA in these organs and tissues, as there are remaining questions regarding the bioavailability and therapeutic efficacy of these preparations, which may be influenced by the structure and composition of the liposome.

The silencing activity of siRNA targeted *Ttr* mRNA (siTTR) in complexes with liposomes was studied by evaluation of the efficiency of *Ttr* mRNA level reduction in the liver of healthy C57BL/6 mice. siTTR/liposome and siScr/liposome complexes at N/P ratio 4/1 were IV administrated to the mice, 7 days after, mice were euthanized, livers were removed, and *Ttr* mRNA levels were measured using real-time RT-PCR.

siTTR complexes with all liposomal formulations demonstrated significant suppression of the *Ttr* mRNA in the liver 7 days after administration compared to the scramble control (p < 0.001, Table 2). However, differences in suppression efficiency could be noted among the tested liposomes. The P800 formulation showed the lowest suppression efficiency (37.5% expression relative to the control) compared to all other liposomal formulations (p < 0.001). The different effects of folate conjugate on P800 and diP800 liposomes may be attributed to structural differences between these lipoconjugates. In the case of P800, the linear PEG structure may provide more flexible positioning of folate for receptor interactions, whereas the branched diP800 structure may create steric hindrance. Additionally, different PEG densities on the surface may lead to receptor saturation effects or competition for binding sites. In contrast, diP2000 and 2 × 3 liposomes demonstrated the highest suppression efficiency (16.7% and 16.9% expression, respectively), with no statistically significant difference between them (p > 0.05). Intermediate suppression efficiencies were observed for diP1500, P2000, P1500, and diP800 formulations (18.2%, 19.1%, 20.7%, and 22.2% expression, respectively). The results show that the structure and molecular weight of lipoconjugates affect the gene silencing activity of siRNA/liposome complexes *in vivo*, with loop-conjugates (diP2000) and reference liposomes (2 × 3) providing the most effective *Ttr* gene silencing in liver.

**TABLE 2:** Silencing activity (% of *Ttr* mRNA level compared to the level in control mice treated with siScr/liposome) of siTTR/liposome after IV administration to C57BL/6 mice. All groups showed statistically significant differences from control (p < 0.001).

Liposome	2 × 3	P800	diP800	P1500	diP1500	P2000	diP2000	Control
*Ttr* mRNA level, % ±SE	16.9 ± 0.2	37.5 ± 1.3	22.2 ± 0.2	20.7 ± 0.2	18.2 ± 0.4	19.1 ± 0.3	16.7 ± 0.4	100 ± 0.3

## 4 Discussion

This comprehensive study investigated various aspects of liposomal delivery systems for nucleic acids, focusing on their structural properties, transfection efficiency, *in vivo* distribution, and gene silencing efficacy. Building on previous research, we utilized 2 × 3-DOPE core system with a 1:1 ratio, aiming to optimize liposome performance for siRNA delivery (Kabilova T. O. et al., 2018). While PEG lipids help prevent undesirable opsonization in the bloodstream, they can also hinder critical steps such as interaction with target cell surfaces and endosomal release (Samaridou et al., 2020). Therefore, we carefully considered PEG content in our formulations, as minimizing PEG content along with increasing ionizable groups has been shown to enhance efficiency fivefold when PEG content is reduced from 10% to ∼1.5 mol% (Semple et al., 2010). The high siRNA binding efficiency of all formulations confirms that the observed differences in transfection activity are due to differences in cellular uptake, endosomal release, or complex stability, rather than inefficient complex formation.

Although higher PEG values and linear structures helped to increase circulation time of complexed siRNA, the best tumor accumulation was observed with diP800, suggesting that compact liposomal structures with specific surface features allow for effective interaction with target cells *in vivo* (Chen et al., 2019; Semple et al., 2010). The relationship between physicochemical properties of liposomes and their biodistribution and tumor-targeting capabilities observed in our study aligns with previous findings (Kabilova T. et al., 2018; Gladkikh et al., 2021).

Our evaluation of antiTTR-siRNA silencing activity in liver tissue revealed that the efficiency of gene silencing is not solely dependent on siRNA delivery quantities but also critically on the ability of siRNA to engage with the RNA-induced silencing complex and effectively silence target genes.

Comparing our findings with other existing siRNA delivery systems reveals several notable distinctions. While FDA-approved siRNA-based drugs like patisiran, givosiran, lumasiran, inclisiran, vutrisiran, and nedosiran primarily utilize bioconjugation for liver-targeted delivery (May Zhang et al., 2021; Adams et al., 2018), our liposomal system offers versatility for targeting different tissues, particularly tumors. The folate-equipped liposomes used in this work and previously (Kabilova T. et al., 2018; Gladkikh et al., 2021) provide a significant advantage over non-targeted delivery systems in tumor-specific accumulation. It is important to note that siRNA biodistribution does not always directly correlate with gene silencing efficiency. This may be attributed to several factors: (1) differences in cellular uptake and endosomal release between different tissues; (2) varying siRNA stability in different tissue microenvironments; (3) tissue-specific factors affecting siRNA processing; (4) different basal expression levels of the target Ttr gene in various tissues. These results underscore the necessity for comprehensive evaluation of both biodistribution and functional activity when developing siRNA delivery systems.

When comparing our liposomal delivery system with those reported in the literature, several advantages become apparent. Commercial lipid nanoparticle formulations typically contain four components: cationic/ionizable lipids, helper lipids, cholesterol, and PEG-lipids (Samaridou et al., 2020, Kulkarni et al., 2018). Our system, based on a single polycationic amphiphile (2 × 3) combined with DOPE and various PEG-lipoconjugates, provides a simpler formulation while maintaining comparable efficacy. The polycationic nature of 2 × 3, with its four protonated amino groups, allows for efficient condensation of siRNA at lower N/P ratios than typically required for conventional cationic lipids, potentially reducing toxicity concerns (Witzigmann et al., 2020; Yin et al., 2014).

Interestingly, our findings on PEG modification paralleled those observed in the delivery of immunostimulatory RNA (isRNA) by Bishani et al. (2023), although with some notable differences. While for isRNA delivery, increasing PEG length (up to 2000 Da) enhanced immunostimulatory activity *in vivo*, our study showed that for siRNA delivery, shorter PEG chains (particularly diP800) provided better tumor accumulation. This difference likely reflects the distinct mechanisms of action of these RNA types: isRNA functions through immune cell activation, where prolonged circulation time is beneficial, while siRNA requires direct cell entry for gene silencing, where compact particle size and effective cellular uptake are more critical (Zatsepin et al., 2016). This suggests that the optimal delivery system design should be tailored to the specific type of RNA cargo and its intended mechanism of action.

Our 2 × 3-DOPE system has demonstrated remarkable versatility beyond siRNA delivery. Previous work by our group has shown its effectiveness for delivery of plasmid DNA (Maslov et al., 2012), antisense oligonucleotides (Kabilova T. et al., 2018), siRNA (Maslov et al., 2012) and mRNA (Vysochinskaya et al., 2022). However, the optimal formulation parameters—such as 2 × 3-to-DOPE ratio and PEG modification—differ depending on the nucleic acid cargo (Chen et al., 2016). For example, while a 1:1 ratio of 2 × 3 to DOPE was optimal for siRNA and isRNA delivery, a 1:3 ratio proved to be superior for mRNA delivery (Vysochinskaya et al., 2022). This highlights the importance of adapting liposomal formulations to the specific properties of the nucleic acid cargo.

The folate targeting approach explored in our study offers particular benefits for tumor-directed therapy. Previously, we demonstrated (Kabilova T. O. et al., 2018; Gladkikh et al., 2021) that folate-equipped 2 × 3-DOPE liposomes significantly enhanced anti-MDR1 siRNA delivery to tumors expressing folate receptors and increased the efficiency of chemotherapy *in vivo*. Our current findings confirm the utility of folate targeting but suggest that the benefits of folate modification may be structure-dependent, with F12 enhancing P800-based delivery but potentially impairing diP800 systems. This complex interplay between targeting ligands and liposome structure warrants further investigation (Kim et al., 2019).

When comparing our delivery system with polymer-based carriers or viral vectors, a number of advantages become apparent, allowing for improved delivery of RNA therapeutics, which is one of the most challenging problems in the field of therapeutic oligonucleotide delivery (Dowdy, 2017). Unlike viral vectors, which pose risks of immunogenicity and insertional mutagenesis, our liposomal system demonstrated minimal toxicity and immunogenicity. Compared to polymer-based systems, which often suffer from complex manufacturing processes and potential toxicity issues, 2 × 3-DOPE liposomal system offers a straightforward preparation method and good biocompatibility. Additionally, the ability to fine-tune surface properties through different PEG modifications provides flexibility not easily achieved with many polymer-based carriers.

A particularly promising application of our delivery system is for combination therapy approaches. Goncharova et al. (2020) demonstrated that 2 × 3-DOPE liposomes modified with P1500 effectively delivered immunostimulatory RNA to limit influenza infection in mice. Combined with our findings on siRNA delivery, this suggests potential for dual delivery of immune-activating isRNA and gene-silencing siRNA—a powerful approach for cancer immunotherapy where both immune activation and suppression of tumor-promoting genes could be achieved simultaneously.

The observed discrepancy between liver accumulation and suppression activity highlights the need to consider not only the amount of siRNA delivered, but also its bioavailability and ability to effectively interact with the cellular machinery, as noted by other researchers (Dammes & Peer, 2020; Gilleron 2013; Setten 2019). The diverse effects of different PEG modifications suggest potential for tailoring delivery systems to specific therapeutic applications—using linear, longer PEG chains when extended circulation is desired, and compact, looped structures when tumor penetration is the priority.

For researchers aiming to employ siRNA in preclinical studies, we recommend the 2 × 3-DOPE (1:1) system as a versatile platform that can be modified with various PEG-lipoconjugates depending on the target tissue. For tumor-targeting applications, the diP800 formulation appears most promising due to its superior tumor accumulation, while for liver-directed therapies, where the highest gene silencing efficacy is required, the diP2000 or unmodified 2 × 3-DOPE systems may be preferable. For studies requiring extended circulation time, P2000 offers advantages, despite potentially lower tissue accumulation. Examining the potential for co-delivery of multiple RNA types (siRNA, mRNA, isRNA) using optimized versions of our delivery system could open new therapeutic avenues. Finally, further refinement of targeting strategies, potentially combining folate with other tumor-specific ligands, might enhance the specificity and efficacy of tumor-directed therapies.

In conclusion, this study serves as a cornerstone for advancing liposomal delivery of nucleic acids. The combination of structural optimization, transfection efficiency analysis, biodistribution studies, and gene silencing evaluation offers a holistic understanding that can accelerate the development of next-generation liposomal therapeutic agents. Our findings demonstrate that the 2 × 3-DOPE platform, with appropriate modifications, represents a versatile and effective system for delivering various types of therapeutic nucleic acids with potential applications ranging from cancer therapy to the treatment of infectious diseases.

## Data Availability

The original contributions presented in the study are included in the article/Supplementary Material, further inquiries can be directed to the corresponding author.
