# Supplementary material for: Effect of structure and composition of cationic liposomes on the delivery of siRNA in vitro and in vivo
Source: Front Pharmacol. 2025 Sep 12;16:1656671. doi: 10.3389/fphar.2025.1656671 (PMC12464033; doi:10.3389/fphar.2025.1656671)
Supplement: Supplementary file 1 [file Supplementaryfile1.docx]

**Supplementary Figure 1.** Structures of cholesterol-based polycationic amphiphile 1,26-bis(cholest-5-en-3-yloxycarbonylamino)-7,11,16,20-tetraazahexacosan tetrahydrochloride (2X3), lipid helper 1,2-dioleoyl-*sn*-glycero-3-phosphoethanolamine (DOPE), lipoconjugates: *O*-(2-(acet)amidoethyl)-*O*′-[2-(*rac*-2,3-di(tetradecyloxy)prop-1-yloxycarbonyl)aminoethyl] octadecaethylene glycol (P800), *O*,*O*’-bis[*rac*-2,3-di(tetradecyloxy) propyl-1-oxycarbonylamino] octadecaethylene glycol (diP800), *O*-(3-(acetamidopropyl)-*O*′-[3-(*rac*-2,3-di(tetradecyloxy)prop-1-yloxycarbonyl)aminopropyl] poly(ethylene glycol_1500_) (P1500), *O*,*O*’-bis[*rac*-2,3-di(tetradecyloxy) propyl-1-oxycarbonylamino] poly(ethylene glycol_1500_) (diP1500), *O-(2-(acetamidoethyl)-O′-[2-(rac-2,3-di(tetradecyloxy)prop-1-yloxycarbonyl)aminoethyl] poly(ethylene glycol_2000_)* (P2000), *O*,*O*’-bis[*rac*-2,3-di(tetradecyloxy) propyl-1-oxycarbonylamino] poly(ethylene glycol_2000_) (diP2000) and *O*-{2-[*rac*-2,3-di(tetradecyloxy) prop-1-yloxycarbonyl]aminoethyl}-*O*’-[2-(pteroyl-L-glutam-5yl)aminoethyl]octadecaethyleneglycol (F12) is used for liposome preparation.


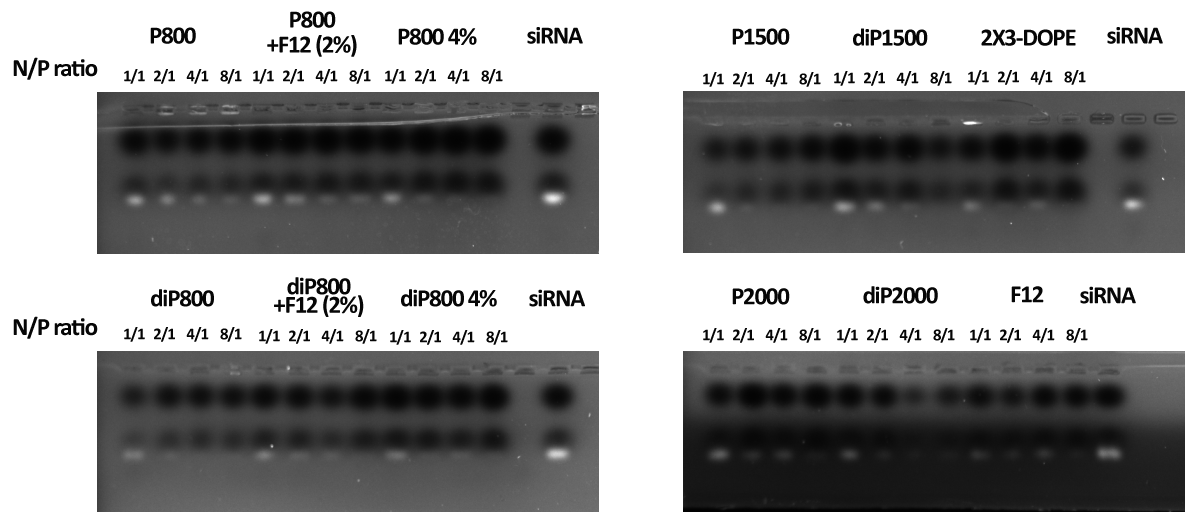


**Supplementary Figure 2.** Evaluation of siRNA binding efficiency to cationic liposomes at various N/P ratios analyzed by gel retardation assay.

Analysis of siRNA complexation with cationic liposomes by agarose gel electrophoresis at different N/P ratios was performed on one gel. Complexes were formed by mixing siRNA (final concentration 0.1 μM) with liposomes at room temperature for 20 min before electrophoresis in 2% agarose gel containing ethidium bromide. Electrophoresis was performed at 120 V for 20 min. Complete siRNA binding was demonstrated by the absence of migration of free siRNA and retention of siRNA-liposome complexes in the loading wells. A progressive decrease in the intensity of the free siRNA band with increasing N/P ratio demonstrates an increase in the efficiency of complexation at higher concentrations of cationic lipids.
